# Association between clusters of back and joint pain with opioid use in middle-aged community-based women: a prospective cohort study

**DOI:** 10.1186/s12891-021-04741-4

**Published:** 2021-10-09

**Authors:** Sultana Monira Hussain, Yuanyuan Wang, Geeske Peeters, Anita E. Wluka, Gita D. Mishra, Helena Teede, Donna Urquhart, Wendy J. Brown, Flavia M. Cicuttini

**Affiliations:** 1 School of Public Health and Preventive Medicine, Monash University, 553 St Kilda Road, Melbourne, VIC 3004 Australia; 2 Department of Geriatric Medicine, Radboud Institute of Health Science, Radboud University Medical Centre, Nijmegen, the Netherlands; 3 Institute for Social Science Research, the University of Queensland, St Lucia, Australia; 4 School of Human Movement and Nutrition Sciences, Faculty of Health and Behavioural Sciences, the University of Queensland, St Lucia, Australia

## Abstract

**Background:**

To determine the relationship between clusters of back pain and joint pain and prescription opioid dispensing.

**Methods:**

Of 11,221 middle-aged participants from the Australian Longitudinal Study of Women’s Health, clusters of back pain and joint pain from 2001 to 2013 were identified using group-based trajectory modelling. Prescription opioid dispensing from 2003 to 2015 was identified by linking the cohort to Pharmaceutical Benefit Scheme dispensing data. Multinomial logistic regression was used to examine the association between back pain and joint pain clusters and dispensing of prescription opioids. The proportion of opioids dispensed in the population attributable to back and join pain was calculated.

**Results:**

Over 12 years, 68.5 and 72.0% women reported frequent or persistent back pain and joint pain, respectively. There were three clusters (‘none or infrequent’, ‘frequent’ and ‘persistent’) for both back pain and joint pain. Those in the persistent back pain cluster had a 6.33 (95%CI 4.38-9.16) times increased risk of having > 50 opioid prescriptions and those in persistent joint pain cluster had a 6.19 (95%CI 4.18-9.16) times increased risk of having > 50 opioid prescriptions. Frequent and persistent back and joint pain clusters together explained 41.7% (95%CI 34.9-47.8%) of prescription opioid dispensing. Women in the frequent and persistent back pain and joint pain clusters were less educated and reported more depression and physical inactivity.

**Conclusion:**

Back pain and joint pain are major contributors to opioid prescription dispensing in community-based middle-aged women. Additional approaches to reduce opioid use, targeted at those with frequent and persistent back pain and joint pain, will be important in order to reduce the use of opioids and their consequent harm in this population.

**Supplementary Information:**

The online version contains supplementary material available at 10.1186/s12891-021-04741-4.

## Background

Chronic pain is complex with a multifactorial aetiology, often starting in early adulthood and with pain prevalence increasing with age [1]. A meta-analysis of 55 studies, including community-based samples who were free from any major condition and were not part of an occupational cohort (i.e. industrial or city worker), reported a 31% prevalence of chronic pain (including back pain and joint pain) in people > 18 years old [2]. Chronic pain decreases work productivity and is associated with billions of dollars in healthcare costs each year [3, 4].

A recent systematic review which included 42 studies (28 from the USA, 9 from European countries, 3 from Canada and 1 each from Australia and India) reported that almost one-third of patients with chronic pain are prescribed opioids [5]. There is significant harm associated with opioid use. For example, long-term use of opioids worsens pain through the development of pain sensitization [6], and prolonged opioid use (defined as at least 1 opioid prescription within the first 90 days after hospital discharge, where the patient was introduced to opioid for the first time [7]) has been associated with high morbidity including opioid use disorders and mortality [8]. There are limited effective methods for managing chronic pain [9]. Thus, in developed countries, compassionate advocacy for better treatment of chronic pain influenced the liberalization of laws governing the opioid prescriptions, and along with aggressive marketing of opioid formulations, resulted in relying on opioids for chronic pain relief [5].

Several recent guidelines, including the Centres for Disease Control and Prevention Guideline for Prescribing Opioids for Chronic Pain, discourage the use of opioid analgesics, especially for the management of some specific pain conditions such as chronic low back pain [10–13]. In order to develop approaches to tackling the current prescription opioid epidemic, understanding those most at risk of chronic opioid use is needed. However, community-based individual-level data are lacking, as previous studies have reported opioid prescribing data from healthcare settings [14–21] or selected populations (e.g. veterans [22], patients with chronic pain [23, 24], or UK biobank (healthy volunteers not representative of UK population) [25]). Furthermore, the prevalence of chronic pain including back pain and joint pain peaks in middle-aged people [26, 27], and in particular middle-aged women are more likely than men to experience chronic pain and tend to report more severe pain than men [28]. Moreover, women were more likely to be prescribed an opioid than men [29]. We aimed to determine the relationship between clusters of back pain and joint pain and prescription opioids dispensing in a cohort of community-based middle-aged women.

## Methods

### Participants

The Australian Longitudinal Study of Women’s Health (ALSWH) is a longitudinal survey of Australian women randomly selected from the Medicare database [30]. The middle-aged cohort born 1946-1951 were recruited in 1996 and re-surveyed in 1998, 2001, 2004, 2007, 2010, and 2013. The surveys included questions about health behaviours, physical and mental health, and sociodemographic factors (www.alswh.org.au). The Human Research Ethics Committees of the University of Newcastle and the University of Queensland approved the study protocol. Written informed consent was obtained from all participants. In the current study we included women who consented to linkage of their ALSWH and Pharmaceutical Benefits Schedule (PBS) record. As data on opioid use was available from 2003 to 2015, women were included from 2001 onwards (Survey 3). All methods were carried out in accordance with relevant guidelines and regulations.

### Demographics and lifestyle factors

Body mass index (BMI): Self-reported height and weight data was used to calculate BMI [30]. BMI was defined by a woman’s weight in kilograms divided by the square of height in meters.

Education: Data on education status was collected only in the 1996 survey. Educational level was grouped as: 1) no formal qualification; school certificate as < 12 years of education; 2) higher school certificate; trade, apprenticeship, certificate or diploma; higher degree or bachelor degree as > 12 years of education.

Physical activity: Participants were asked to only report activity that lasted 10 min or more in 1) walking briskly, 2) moderate leisure activity i.e. recreational swimming, dancing, social tennis, 3) vigorous leisure activity that makes you breathe harder or puff and pant [31]. A physical activity score was calculated as the sum of the products of total weekly minutes in each of the three categories of activity and the metabolic equivalent value (MET) assigned to each category. Physical activity was then categorised based on total MET.minutes per week. Inadequate physical activity was defined by < 600 MET·minutes per week (150 min moderate-intensity physical activity/week) [31].

Depression: Depression was assessed by the question “In the past three years, have you been diagnosed or treated for depression” [32].

### Back pain and joint pain

#### Back pain

Women were asked “In the last 12 months have you had back pain?” They were asked to circle one response: ‘never’, ‘rarely’, ‘sometimes’ or ‘often’. Participants who responded ‘never’ or ‘rarely’ were categorized as having ‘no back pain’, while those who responded ‘sometimes’ or ‘often’ were categorized as ‘having back pain’ [33].

#### Joint pain

Participants were asked, “In the last 12 months have you had stiff or painful joint?” Those who responded ‘never’ or ‘rarely’ in response to this question were categorized as having ‘no joint pain’, and those who responded ‘sometimes’ or ‘often’ were categorized as having joint pain.

#### Clusters of back pain and joint pain

We categorised back pain and joint pain into different clusters using the group-based trajectory modelling (GBTM) and identified participants who followed a similar back pain or joint pain pattern over time [34]. This provided a categorical variable describing the clusters of back pain and clusters of joint pain [34–36].

### PBS items for opioids

The PBS Schedule lists all the medicines available to be dispensed to patients at a government-subsidised price. The PBS data provide dispensing records of medication including the date of dispensing and the PBS item code that provides information on the medication name, formulation, strength and type of script. PBS item codes are specific for each generic type of drug, dose and number of units per container. Most of the PBS listed medicines are dispensed by pharmacists, and used by patients at home. PBS is available to all Australian residents who hold a current Medicare card. However, private prescriptions are not included in the PBS dataset. PBS website (http://www.pbs.gov.au) was used to identify the item codes for tramadol, oxycodone, combined oxycodone and naloxone (Targin), buprenorphine, codeine, codeine combined with panadol (panadiene), morphine, fentanyl, hydromorphone, and methadone. PBS data for 2014 and 2015 were not complete, as there is a time lag in PBS collecting dispensing data from public hospitals and in people claiming medication reimbursement if they have paid the original prescription during this time [37].

Based on the total number of prescriptions dispensed over the entire 12 year study period, women were grouped into 5 categories (1) no prescriptions, (2) 1-15 prescriptions, (3) 16-50 prescriptions, (4) > 50 prescriptions. Based on the maximal number of prescriptions prescribed in any year over the 12 years (‘number of prescriptions/year’), women were grouped into three categories (1) no prescriptions in any calendar year, (2) 1 prescription in at least one year, and (3) ≥2 prescriptions in at least one year. The categories were chosen based on the evidence that the probability of long-term opioid use increases significantly with as little as 5 days of use [38], irrespective of the reason of prescribing, including surgery.

### Statistical analysis

#### Identification of clusters of back pain and joint pain

We used GBTM to identify pain clusters of participants, who have followed a similar back pain or joint pain pattern over time [34]. The statistical details of the methodology in the context of biomedical and sociological research have been described in detail elsewhere [36, 39]. We used the data from 2001 as baseline and the subsequent follow-ups until 2013 (2001, 2004, 2007, 2010 and 2013) for these analyses. We conducted GBTM analysis using STATA TRAJ [40]. A series of models using all available data to estimate latent clusters with linear and quadratic terms for each group was systematically fitted and compared using the Bayesian Information Criterion (BIC) statistic. The choice of the number of clusters was therefore guided by the goal of analysis and confirmation of model adequacy, and the proportion of cohort members in each class [41].

#### Back pain, joint pain and opioid prescription dispensing

There are no missing data for back pain and joint pain as GBTM handles missing data by fitting the model using maximum likelihood estimation [34]. We included 11,221 participants who completed Survey 3 (March 2001-2002).

General characteristics of the women placed in different clusters of back pain and joint pain were compared using ANOVA for continuous variables and chi-square for categorical variables. Number of women prescribed opioid prescriptions over the study period (12 years) and ‘number of prescriptions/year’ were compared using chi-square. Types of opioid prescriptions according to back pain and joint pain clusters and their morphine equivalences were described. Multinomial logistic regression was used to examine the association between back pain and joint pain clusters and prescription opioid dispensing using the no or infrequent back pain, no or infrequent joint pain; and no opioid prescriptions as the referent category. Population attributable fraction (PAF) was calculated to determine the proportion of opioid prescription dispensed in the population that could be attributable to back pain or joint pain using the “punafcc” command in Stata, which implements the method recommended by Greenland and Drescher [42]. The formula for PAF used is∑pOp[(RR − 1)/RR], where pOp is the proportion of total opioid prescription dispensing observed in the participants who had back pain only, or joint pain only, or either back or joint pain and RR is the relative risk associated with either back pain only, joint pain only, or either back or joint pain. The PAFs represent the proportion of opioid prescriptions dispensing that would not have occurred if members of that frequent and persistent back pain or joint pain category were redistributed to lowest risk cluster (no or infrequent back pain, no or infrequent joint pain, no or infrequent back or joint pain), assuming that the observed associations represent causal effects. Model 1 was unadjusted analyses, and model 2 was adjusted for age, BMI, education, physical activity, and depression. Confounders were selected based on previously published studies examining opioid use in the back pain and joint pain areas, using the directed acyclic graph (DAG) diagram [43] (Supplementary Fig. 1). All analyses were performed using Stata SE version 16.0 (StataCorp).

## Results

We identified three clusters for each of back pain and joint pain. For back pain, 3527 (31.4%) were in the ‘no to infrequent’ (pain reported in 0-2 of 5 surveys, with 3386 (96.0%) having pain 0-1 survey), 3493 (31.1%) in ‘frequent’ (pain reported in 3-4 of 5 surveys), and 4201 (37.4%) in the ‘persistent’ (pain reported at all 5 surveys) back pain cluster. Regarding joint pain, 3141 (28.0%) were in the ‘no to infrequent’ (pain reported in 0-2 of 5 surveys, 3015 (96.0%) having pain 0-1 survey), 3376 (30.1%) were in the ‘frequent’ (pain reported in 3-4 of 5 surveys), and 4704 (41.9%) in ‘persistent’ (pain reported at all 5 surveys) cluster. Opioid prescriptions were dispensed to 5126 participants over the study period (2003-2015). Of the 5126 women, 92.1% had one prescription/year and the rest had > 2 prescriptions/year.

Demographic characteristics and number of opioids prescriptions dispensed (over the study period, and ‘number of prescriptions/year’) based on back pain and joint pain cluster are presented in Table 1. For both back pain and joint pain, women in the ‘persistent’ pain cluster were of higher BMI, less educated, more likely to have depression and be inactive than women in the other two clusters. The number of opioid prescriptions dispensed over the study period and ‘number of prescriptions/year’ were highest in women in the ‘persistent’ followed by the ‘frequent’ pain clusters for both back pain and joint pain. Types of opioids in these prescriptions and the morphine dose per script (mg) are shown in Supplementary Table 1.

**Table 1:** Demographic and psychosocial characteristics and opioid prescriptions middle-aged community-based women from 2003 to 15 (*N* = 11,221)

	No or infrequent pain	Frequent pain	Persistent pain	p
Back pain (n, %)	3527 (31.4)	3493 (31.1)	4201 (37.4)	
Age in years^a^	52.0 (1.5)	52.0 (1.5)	52.1 (1.4)	< 0.02
BMI, kg/m^2a^	26.0 (5.0)	26.9 (5.4)	27.5 (5.9)	< 0.001
Education (Did not complete high school, < 12 years education)^a^	2012 (57.5)	1786 (51.7)	1926 (46.1)	< 0.001
Depression^a^	246 (7.1)	376 (10.9)	639 (15.4)	< 0.001
Inadequate physical activity^a^	750 (22.3)	849 (25.9)	1058 (26.9)	< 0.001
Number of women dispensed the following no of prescriptions 2003-2015				< 0.001
None (*n* = 6095)	2290 (64.9)	1905 (54.5)	1900 (45.2)	
1-15 prescriptions (*n* = 4272)	1141 (32.4)	1359 (38.9)	1772 (42.2)	
16-50 prescriptions (*n* = 483)	60 (1.7)	147 (4.2)	276 (6.6)	
> 50 (*n* = 371)	36 (1.0)	82 (2.4)	253 (6.0)	
‘Number of prescriptions/year’				< 0.001
No prescriptions (n = 6095)	2290 (64.9)	1905 (54.5)	1900 (45.2)	
1 prescription (*n* = 4721)	1202 (34.1)	1481 (42.4)	2038 (48.5)	
2 or more prescription (*n* = 405)	35 (1.0)	107 (3.1)	263 (6.3)	
Joint pain (n)	3141 (28.0)	3376 (30.1)	4704 (41.9)	
Age in years^a^	52.3 (1.3)	52.1 (1.5)	51.8 (1.5)	< 0.001
BMI, kg/m^2a^	25.5 (4.7)	26.6 (5.2)	27.9 (5.9)	< 0.001
Education (Did not complete high school, < 12 years education)^a^	1372 (44.1)	1604 (47.8)	2432 (52.1)	
Depression^a^	207 (6.7)	332 (9.9)	722 (15.5)	< 0.001
Inadequate physical activity^a^	664 (22.3)	804 (25.2)	1189 (27.0)	< 0.001
Number of women dispensed the following no of prescriptions 2003-2015				< 0.001
No prescriptions (n = 6095)	2071 (65.9)	1891 (56.0)	2133 (45.3)	
1-15 prescriptions (n = 4272)	982 (31.3)	1296 (38.4)	1994 (42.4)	
16-50 prescriptions (n = 483)	57 (1.8)	123 (3.6)	303 (6.4)	
> 50 prescriptions (n = 371)	31 (1.0)	66 (2.0)	274 (5.8)	
‘Number of prescriptions of opioids/year’				< 0.001
No prescription (n = 6095)	2071 (65.9)	1891 (56.0)	2133 (45.3)	
1 prescription (n = 4721)	1031 (32.8)	1405 (41.6)	2285 (48.6)	
2 or more prescriptions (n = 405)	39 (1.2)	80 (2.4)	286 (6.1)	

^a^Baseline data presented (collected in 2001 survey), continuous variables are presented as mean (±SD)

The association between back pain and joint pain clusters, and opioid prescription dispensing is presented in Table 2. For opioid prescription dispensing over the 12 years, an increasing number of opioid prescriptions was dispensed in the ‘frequent’ and ‘persistent’ back pain clusters relative to the ‘no or infrequent’ cluster. After adjustment for age, BMI, education, physical activity and depression, compared to those in the ‘no or infrequent’ back pain cluster, women in the frequent back pain cluster had increased risk of opioid prescriptions dispensing: relative risk ratio (RRR) 1.34 (95% CI 1.21-1.49) for dispensing 1-15 prescriptions, RRR 2.52 (95% CI 1.83-3.48) for 16-50 prescriptions, and RRR 2.20 (95% CI 1.45-3.33) for > 50 prescriptions. Persistent back pain also increased the risk of opioid prescriptions dispensing: RRR 1.66 for 1-15 prescriptions, RRR 4.34 for 16-50 prescriptions, and RRR 6.33 for > 50 prescriptions. Similar increased risk for opioid prescription dispensing was observed for joint pain clusters. The frequent and persistent back pain and joint pain clusters were associated with a higher ‘number of prescriptions/year’ dispensed than the ‘no or infrequent’ cluster (Supplementary Table 2).

**Table 2:** Relationship between musculoskeletal pain and opioid prescriptions over the study period (2003-15)

	Model 1 RRR (95% CI)			Model 2 RRR (95% CI)		
	1-15 prescriptions vs no prescription	16-50 prescriptions vs no prescription	> 50 prescriptions vs no prescription	1-15 prescriptions vs no prescription	16-50 prescriptions vs no prescription	> 50 prescriptions vs no prescription
Back pain						
No or infrequent back pain	Reference	Reference	Reference	Reference	Reference	Reference
Frequent back pain	1.43 (1.30, 1.58)	2.95 (2.17, 4.00)	2.74 (1.84, 4.07)	1.34 (1.21, 1.49)	2.52 (1.83, 3.48)	2.20 (1.45, 3.33)
Persistent back pain	1.87 (1.70, 2.06)	5.54 (4.17, 7.38)	8.47 (5.94, 12.07)	1.66 (1.50, 1.84)	4.34 (3.22, 5.86)	6.33 (4.38, 9.16)
Joint pain						
No or infrequent joint pain	Reference	Reference	Reference	Reference	Reference	Reference
Frequent joint pain	1.44 (1.30, 1.60)	2.36 (1.72, 3.26)	2.33 (1.51, 3.59)	1.42 (1.27, 1.58)	2.16 (1.55, 3.02)	2.03 (1.30, 3.15)
Persistent joint pain	1.97 (1.79, 2.17)	5.16 (3.86, 6.89)	8.58 (5.89, 12.50)	1.85 (1.67, 2.06)	4.23 (3.11, 5.76)	6.19 (4.18, 9.16)

Data are presented as relative risk ratio (RRR) and 95% confidence intervals (CI). Model 1: unadjusted; Model 2: adjusted for age, BMI, education, physical activity and depression

PAF was calculated to examine the contribution of back pain and joint pain to the opioid prescriptions dispensed over the 12 years (Table 3). The PAF for opioid prescription dispensing was 30.4% for back pain and 35.1% for joint pain, adjusted for age, BMI, education, physical activity and depression. Back and joint pain together explained 41.7% of opioid prescriptions.

**Table 3:** Population attributable fraction (PAF, %) of opioid use in relation to back and joint pain

	Model 1 PAF (95% CI)	Model 2 PAF (95% CI)
Back pain (No or infrequent back pain vs frequent and persistent pain)	35.8 (31.9, 39.4)	30.4 (25.9, 34.6)
Joint pain (No or infrequent joint pain vs frequent and persistent pain)	38.6 (34.5, 42.4)	35.1 (30.4, 39.4)
Back pain or joint pain (No or infrequent pain vs frequent and persistent pain)	49.2 (43.7, 50.1)	41.7 (34.9, 47.8)

Data are presented as population attributable risk fraction (PAF) and 95% confidence intervals (CI). Model 1: unadjusted; Model 2: adjusted for age, BMI, education, physical activity and depression

## Discussion

In this cohort of community-based middle-aged women followed for 12 years, more than two thirds of women were in the frequent or persistent back pain clusters (pain reported in > 3 out of 5 surveys). Similarly, over 70% of women were in the frequent or persistent joint pain clusters (pain reported in > 3 out of 5 surveys). These clusters for both back pain and joint pain were associated with higher number of opioid prescriptions dispensing over the study period and yearly prescription dispensing (‘prescriptions/year’). Frequent and/or persistent back pain attributed to 30.4% of the opioid prescription dispensing and frequent and/or persistent joint pain attributed to 35.1% of the opioid prescription dispensing, together explaining 41.7% of the prescription opioid dispensing.

Our findings of distinct back pain clusters over 12 years are supported by previous studies with three [44], five [45] and seven [46] years of follow-up, suggesting that back pain is often chronic. We also found three distinct clusters for joint pain with 41.9% of middle-aged women with persistent joint pain over 12 years. This has not previously been reported in community-based populations. However, one recent study examined pain clusters across several body sites in 1336 adolescents (11–14 years old) over three years and found that 1–5% of adolescents were in the ‘persistent’ pain cluster [47].

We found that women in the frequent and persistent back pain clusters and the frequent and persistent joint pain clusters were dispensed a significantly higher number of opioid prescriptions over the study period and a higher rate of yearly opioid prescriptions. For example, those in the persistent back pain cluster had a more than 6-fold increased risk of having > 50 opioid prescriptions over the 12 years. This is similar to the result reported from the UK biobank, where 5.5% people with chronic musculoskeletal pain were using opioids on a regular basis [25]. Most commonly used prescription contain 20 to 60 units of opioids [29]. These results are in line with the reported over-prescription of opioids for chronic pain, including musculoskeletal pain in several developed countries including UK, USA, France, Canada, Norway and Australia [14–21] and extend these data by examining the opioid dispensing based on the presence of pain clusters over 12 years. The results from the UK biobank showed that 5.5% people with chronic musculoskeletal pain were using opioids on a regular basis [25]. Another study from the UK reported prescribed opioids in 59% of participants with chronic musculoskeletal pain [14]. In the US survey, 18.8% of people who were suffering from chronic back pain used opioids [48]. The use of opioids for back pain and joint pain in emergency department settings remains variable. For example, a study from Canada, including participants with non-urgent low back pain over a six-year period, showed that one quarter of the participants were prescribed hydromorphone [49]. In another study, 51% of the opioid naïve back pain patients received opioids in the emergency department [50]. Our study extends the understanding beyond these studies by reporting opioid use in a community-based sample in relation to back pain and joint pain clusters over 12 years. Our findings support the notion that chronic opioid use fails to achieve adequate long-term pain relief [51]. Of concern is the increasing evidence that long-term opioid use may further aggravate chronic pain through the development of pain sensitization [6].

In our study, 1180 women (10.5% of the total population) in the frequent or persistent back pain clusters and 1240 (11.1% of the total population) in the frequent or persistent joint pain clusters were dispensed prescriptions containing ≥400 mg morphine equivalence of opioid for use at least 20 days (Supplementary Table 1). This is troublesome as there is evidence that even opioid naïve patients who receive a cumulative dose of ≥400 mg (versus < 120 mg) morphine equivalence in their first month of therapy are 2.96 times more likely to become chronic opioid users [52].

We found that frequent and/or persistent back pain and joint pain attributed to 41.7% of opioid dispensing in this population. This calls for increasing awareness that musculoskeletal pain is a major reason for prescription of opioids. For example, in the CONsortium to Study Opioid Risks and Trends which included adults aged > 18 years and in the Group Health and Kaiser Permanente of Northern California, of those prescribed long-term opioids, 29.9 and 27.2% of participants reported back pain, respectively [53]. In this study, we cannot directly attribute opioid dispensing to back pain or joint pain. However, we found that the opioid dispensing was common and use was chronic. There is evidence that those with chronic pain commonly have pain at more than one site, but with one site predominating [54]. Management of chronic low back pain and joint pain remains poor which is associated with multisite pain [55]; a multidisciplinary approach is needed [10, 11, 13] for improved pain management. Women with lower socioeconomic status are less likely to have access to exercise, lifestyle and psychological programs for managing musculoskeletal pain and preventing the development of chronic pain. Our data showed that those prescribed opioids were less educated and reported less physical activity and more depression, raising the concern that there may be an over reliance on opioids to manage the chronic pain in this group. This has policy implications for the development of viable strategies for dealing with chronic musculoskeletal pain, and for countering the current prescription opioid epidemic. This should include but not be limited to: 1) identifying people with chronic musculoskeletal pain who are prone to get opioid prescriptions; 2) examining pre-existing policies for managing pain, and enablers and barriers to implementing these strategies; 3) encouraging general practitioners not to prescribe opioids, but rather educate patients lifestyle modifications, as per the current guidelines. Our data suggest that women with chronic back pain and joint pain need to be identified as being at high risk of chronic opioid use and strategies should be developed to reduce the prescription opioid dispensing. Guidelines for the management of low back pain recommend education regarding management of pain, management of psychological issues, encouragement to participate in physical activity and advice to return to normal activity [56]. Only simple analgesics should be prescribed and if needed, opioids should be prescribed with caution [56].

Potential limitations of this study are that sociodemographic and pain data were self-reported [33]. We do not have data on other co-morbidities for which opioids were used. However, we have followed up this community-based sample of women over a 12 years period. It is less that these women were prescribed opioids for such a long time for other significant medical conditions such as cancer. We cannot conclude that these women were prescribed opioids specifically for back pain or joint pain. However, although those with back pain and joint pain commonly have other chronic pain problems such as headaches or abdominal pain [57], opioids are not generally recommended for these conditions. Furthermore, data suggest that more than 50% of opioid prescriptions are issued to people with low back pain [20]. These results in the middle-aged women are of particular interest since use of opioids increased with age-group and opioid use is more common in females than males [25, 28] The PBS provides near-complete coverage of prescription medicine dispensing in Australia. There is evidence that PBS claims under-estimate opioid use in Australia [29]. For example in the years, 2010, 2011 and 2014 pharmaceutical claims data did not account for 19.1, 20.0 and 12.4% of prescription-only opioid utilisation respectively [29]. Although total opioid use in an individual may be underestimated, all Australian citizens and permanent residents can access medications listed on the PBS with no restrictions. Although not a direct measure of medication utilization, prescriptions are a good indicator of overall opioid use [18]. PBS does not take into account the over the counter opioid use. Codeine was the only opioid available over the counter in Australia until 2018, so our data underestimates total codeine use. However, our study is set in the context of the Australian healthcare system where there is universal health cover, so everyone has access to medications through the PBS. Thus, there is no barrier to accessing a general practitioner or other health professional who can prescribe opioid if considered necessary for a patient. So it is unlikely that there is significant bias in those taking over-the-counter codeine being very different to those who have prescription opioids, but we may have underestimated the total number of doses of opioids. In women where prescriptions were initiated in a public hospital, most will have had an ongoing prescription through their general practitioners and PBS. Although we have access to PBS data until 2015, the trend in opioid use in Australia has remained similar since then as recently reported (November 2018) [29]. Strengths of this study include the large, well characterized cohort of middle-aged women largely representative of community dwelling Australian women, unselected in relation to pain or healthcare seeking behaviour, the availability of individual person-level data, long duration of follow-up, and reliable data on opioid prescriptions obtained from the PBS database.

## Conclusions

In conclusion, women in the frequent and persistent back pain and/or joint pain clusters were prescribed substantial amounts of opioids, including high dose opioids, which make the women vulnerable to chronic opioid use, misuse, and overdose. More than 40% of opioid prescriptions that were dispensed could be attributed to the presence of frequent and/or persistent back and joint pain. Additional approaches to reduce opioid use, targeted at those with back pain and joint pain, will be important in order to reduce the use of opioids and their consequent harm in this population.

## Supplementary Information

**Additional file 1: Supplementary Table 1**: Type of opioid prescribed in middle aged community based women from 2003 to 15.

**Additional file 2: Supplementary Table 2**: Relationship between musculoskeletal pain and yearly opioid prescriptions.

**Additional file 3: Supplementary Fig. 1**: The relationship of back and joint pain, and the confounders with opioid use.

## Data Availability

The datasets used and/or analysed during the current study are available from the corresponding author on reasonable request.

## Abbreviations

ALSWH: Australian Longitudinal Study of Women’s Health
PBS: Pharmaceutical Benefits Schedule
BMI: Body mass index
GBTM: Group-based trajectory modelling
BIC: Bayesian Information Criterion
PAF: Population attributable fraction
RRR: Relative risk ratio
CI: Confidence interval
