# Supplementary material for: Association between clusters of back and joint pain with opioid use in middle-aged community-based women: a prospective cohort study
Source: BMC Musculoskelet Disord. 2021 Oct 9;22:863. doi: 10.1186/s12891-021-04741-4 (PMC8502269; doi:10.1186/s12891-021-04741-4)
Supplement: Supplementary file 1 — Additional file 1: Supplementary Table 1: Type of opioid prescribed in middle aged community based women from 2003 to 15. [file 12891_2021_4741_MOESM1_ESM.docx]

**Supplementary Table 1: Type of opioid prescribed in middle aged community based women from 2003-15**

| **Types of opioid** | **Number of units/script** | **Morphine dose per script (mg)** |
| --- | --- | --- |
| Tramadol | 20 | 100-600 |
| Oxycodone | 20-28 | 280-1600 |
| Buprenorhine | 1-2 | 140-280 |
| Codeine | 20-60 | 78-234 |
| Morphine | 28 | 840 |
| Fentanyl | 5 | 540-1080 |
| Hydromorphone and methadone | 10 | 200-800 |
