# Supplementary material for: Association between clusters of back and joint pain with opioid use in middle-aged community-based women: a prospective cohort study
Source: BMC Musculoskelet Disord. 2021 Oct 9;22:863. doi: 10.1186/s12891-021-04741-4 (PMC8502269; doi:10.1186/s12891-021-04741-4)
Supplement: Supplementary file 2 — Additional file 2: Supplementary Table 2: Relationship between musculoskeletal pain and yearly opioid prescriptions. [file 12891_2021_4741_MOESM2_ESM.docx]

**Supplementary Table 2: Relationship between back pain and joint pain, and yearly opioid prescriptions**

|  | Model 1  RRR (95% CI) | | Model 2  RRR (95% CI) | |
| --- | --- | --- | --- | --- |
|  | 1 prescription  versus no prescription | 2 or more prescription  versus no prescription | 1 prescription  versus no prescription | 2 or more prescriptions  Versus no prescription |
| Back pain |  |  |  |  |
| No or infrequent back pain | Reference | Reference | Reference | Reference |
| Frequent back pain | 1.48 (1.34, 1.63) | 3.67 (2.50, 5.41) | 1.37 (1.24, 1.52) | 3.01 (2.01, 4.51) |
| Persistent back pain | 2.04 (1.86, 2.24) | 9.06 (6.33, 12.95) | 1.78 (1.61, 1.97) | 6.72 (4.63, 9.76) |
| Joint pain trajectories | | | | |
| No or infrequent back pain | Reference | Reference | Reference | Reference |
| Frequent back pain | 1.49 (1.34, 1.65) | 2.25 (1.52, 3.31) | 1.45 (1.30, 1.61) | 2.23 (1.47, 3.36) |
| Persistent back pain | 2.15 (1.96, 2.37) | 7.12 (5.07, 10.00) | 1.97 (1.78, 2.19) | 6.12 (4.22, 8.86) |

Data are presented as relative risk ratio (RRR) and 95% confidence intervals (CI). Model 1: unadjusted; Model 2: adjusted for age, BMI, education, physical activity and depression
