# Supplementary figures and images for: Association between clusters of back and joint pain with opioid use in middle-aged community-based women: a prospective cohort study
Source: BMC Musculoskelet Disord. 2021 Oct 9;22:863. doi: 10.1186/s12891-021-04741-4 (PMC8502269; doi:10.1186/s12891-021-04741-4)

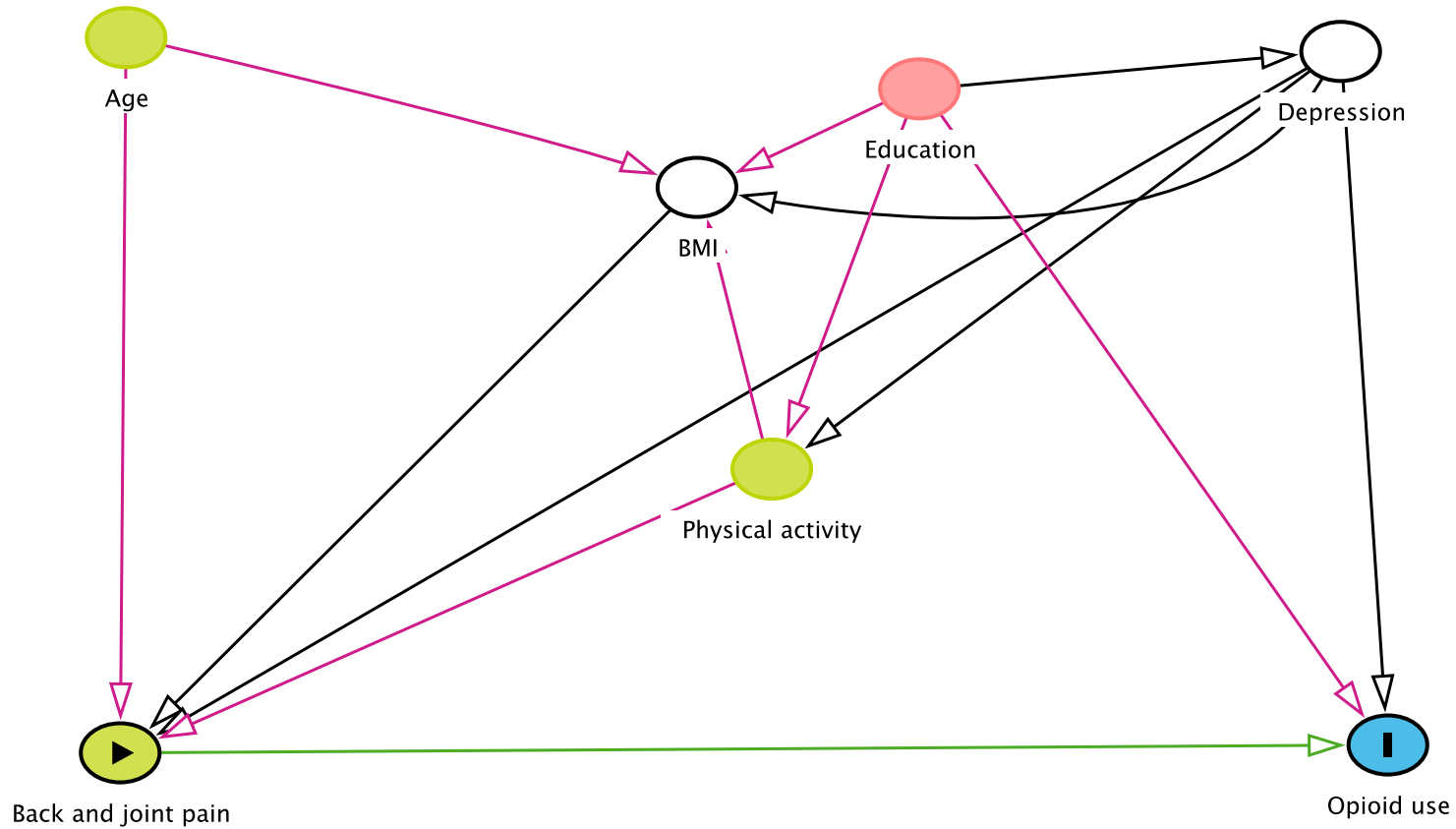

Supplement: Supplementary file 3 — Additional file 3: Supplementary Fig. 1: The relationship of back and joint pain, and the confounders with opioid use. [file 12891_2021_4741_MOESM3_ESM.pdf]
